# Impact of BRAF, MLH1 on the incidence of microsatellite instability high colorectal cancer in populations based study

**DOI:** 10.1186/1476-4598-7-68

**Published:** 2008-08-21

**Authors:** Hassan Brim, Pooneh Mokarram, Fakhraddin Naghibalhossaini, Mehdi Saberi-Firoozi, Mansour Al-Mandhari, Kamla Al-Mawaly, Rayhaneh Al-Mjeni, Abeer Al-Sayegh, Sandy Raeburn, Edward Lee, Francis Giardiello, Duane T Smoot, Alexander Vilkin, C Richard Boland, Ajay Goel, Mitra Hafezi, Mehdi Nouraie, Hassan Ashktorab

**Affiliations:** 1 Department of Medicine and Cancer Center, Howard University, Washington, D.C, USA; 2 Biochemistry Department, Shiraz University Medical Sciences, Shiraz, Iran; 3 Gastroentrology Division, Shiraz University Medical Sciences, Shiraz, Iran; 4 Department of Medicine, Sultan Qaboos University, Muscat, Oman; 5 Department of Pathology, Howard University, Washington, D.C, USA; 6 Department of Medicine, The Johns Hopkins University, Baltimore, Maryland, USA; 7 Department of Medicine and Cancer Center, Baylor University Medical Center, Dallas, Texas, USA

## Abstract

We have identified an alternative pathway of tumorigenesis in sporadic colon cancer, involving microsatellite instability due to mismatched repair methylation, which may be driven by mutations in the BRAF gene (V600E). Colorectal cancer (CRC) is the most common cancer in the world, and African Americans show a higher incidence than other populations in the United States. We analyzed sporadic CRCs in Omani (of African origin, N = 61), Iranian (of Caucasian origin, N = 53) and African American (N = 95) patients for microsatellite instability, expression status of mismatched repair genes (hMLH1, hMSH2) and presence of the BRAF (V600E) mutation. In the Omani group, all tumors with BRAF mutations were located in the left side of the colon, and for African Americans, 88% [7] of tumors with BRAF mutations were found in the right side of the colon. In African Americans, 31% of tumors displayed microsatellite instability at two or more markers (MSI-H), while this rate was 26% and 13% for tumors in the Iranian and Omani groups, respectively. A majority of these MSI-H tumors were located in the proximal colon (right side) in African American and Iranian subjects, whereas most were located in the distal colon (left side) in Omani subjects. Defects in hMLH1 gene expression were found in 77% of MSI-H tumors in both African Americans and Iranians and in 38% of tumors in Omanis. BRAF mutations were observed in all subjects: 10% of tumors in African Americans (8/82), 2% of tumors in Iranians (1/53), and 19% of tumors in Omanis (11/59). Our findings suggest that CRC occurs at a younger age in Omani and Iranian patients, and these groups showed a lower occurrence of MSI-H than did African American patients. Our multivariate model suggests an important and significant role of hMLH1 expression and BRAF mutation in MSI-H CRC in these populations. The high occurrence of MSI-H tumors in African Americans may have significant implications for treatment, since patients with MSI-H lesions display a different response to chemotherapeutic agents such as 5-fluorouracil.

## Introduction

Colorectal cancer (CRC) is the second leading cause of cancer-related deaths in the United States, despite recent improvements in the diagnosis and treatment of the disease [1]. The incidence and mortality rate of CRC are higher in African Americans than in the general population [1,2]. Many epidemiological and genetic investigations have focused on African Americans [2-4] with the goal of deciphering the reasons for such disparities. While socio-economic factors are likely involved (e.g., African Americans tend to reach more advanced stages of disease before diagnosis), biological factors also contribute to the disparity [5]. Dietary and environmental factors certainly play an important role, as Japanese immigrants to the US show higher colon cancer rates than do Japanese people living in Japan. The latter also started showing higher rates of colon cancer along with the westernization of their diets [6,7].

The recent approval by the FDA of Bidil, a drug for heart failure in African Americans, illustrates ethnical/racial genetic specificities that lead to different interactions with a given drug or molecule [8]. Only limited genetic and epigenetic studies exist from Africa and other continents, including Asia, that have populations known to be of African descent. In Oman, some tribes have Bedouin roots and others have Asian and/or African origins [9]. In general, the Omani population has Asian and African ethnicities [10]. Other ethnic groups in Asia are not considered to be of African descent, such as Caucasian people in Iran. In this study, we used samples from patients treated for CRC in Oman, Iran and the US (African Americans) for genetic and epigenetic investigation. We chose these three populations based on their similarities and differences in African ethnicity [10-14].

Colon cancer develops through different pathways, all involving changes at the chromosomal or genetic levels. Other modifications occur epigenetically and affect the level of expression of certain targeted genes that are essential for the normal control of cell division within the colon mucosa [15]. It is now widely accepted that sporadic colorectal cancers frequently arise from pre-neoplastic lesions through the activation of oncogenes (K-ras, BRAF) and the inactivation of tumor suppressor genes (APC, p53, DCC) and mismatch repair (MMR) genes (MLH1 and MSH2) [16,17]. Also, activating mutations in BRAF, one of the RAF genes which encode kinases that are regulated by Ras and mediate cellular responses to growth signals, have been found to be associated with MSI-H cancers [18]. The DNA MMR process can be impeded by genes that are either mutated [19] or silenced [4], leading to the expression of non-functional proteins or to a lack of expression, respectively. MMR pathway is primarily responsible for colon cancer development in families with Hereditary Non Polyposis Colon Cancer (HNPCC), which represents about 5% of the generally inventoried colon cancer cases [20].

In the present study, we analyzed samples from two different populations of African descent (African American and Omani) and a Caucasian population (Iranian) for microsatellite instability and BRAF mutations. We correlated our findings with the silencing of genes involved in DNA MMR. We evaluated and compared the demographic and clinicopathological data in relation to molecular alterations in specific genes (BRAF, DNA MMR) to improve our understanding of colon tumorgenesis in people living in different environments.

## Methods

### Patients

A total of 95 African American CRC patients (from Howard University and Johns Hopkins Hospital between 1997 and 2004; self-identified as African American), 61 Omani CRC patients (from the two tertiary referral hospitals in Oman Sultan Qaboos University Hospital and the Royal Hospital between 2000 and 2004 who underwent surgical resection) and 53 Iranian CRC patients (from the two referral hospitals in Shiraz, Iran Namazi Hospital and Saadi Hospital) were included in this study. Formalin-fixed, paraffin-embedded archival tissue was collected (with approval from all above sites' Institutional Review Boards) and clinical data was obtained (including race, age, site of primary tumor, mucin production and tumor differentiation). In addition, the family history of cancer was analyzed to identify those pedigrees that met either the Amsterdam I or Amsterdam II criteria for HNPCC. The study was performed on unselected and serially collected specimens. All cases underwent surgery within the given collection.

### DNA isolation and MSI analysis

Archived and fresh tumor blocks were cut into 5-μm sections on Superfrost slides (Fisher Scientific, Pittsburgh, PA). The tumor and normal area were diagnosed by a pathologist using the H&E matched slide and microdissected to pinpoint the tumor areas as well as normal areas from at least two slides. A pinpoint DNA extraction kit was used as recommended by the manufacturers (Zymo Research). The extracted tumor and normal matched DNA were used as template in PCR reactions where five microsatellite markers (Cancer Research 58:5248–5257, 1998;[21]; BAT25, BAT26, D17S250, D5S346 and D2S123) were used to evaluate MSI status. PCR products were analyzed in a 3130 ABI GeneScan. Those displaying DNA instability at only one of the markers (including the dinucleotides) were labeled MSI-L, those displaying instability with two or more markers were labeled MSI-H, and those displaying no instability with any of the five markers tested were labeled MSS. Due to unclear characteristics of MSI-L, we combined MSS and MSI-L as one group (non-MSI-H).

### Methylation-specific PCR

The presence or absence of hMLH1 and p16 methylation in cancers was determined by comparing the signals from tumor-derived tissues with those from normal, non-cancerous tissues, as previously described [3,4]. DNA was extracted from the samples using a Gentra DNA extraction kit (Gentra) and was modified using DNA modification kits as recommended by the manufacturers (Zymo Research). MSP was used to distinguish unmethylated from methylated alleles, based on sequence alterations produced by bisulfite treatment of DNA, to convert unmethylated cytosines to uracil. These changes are identified by subsequent PCR using primers specific to the methylated (unchanged) or unmethylated (changed) DNA. Briefly, 1 μg of genomic DNA was denatured by treatment with NaOH and was modified by the addition of sodium bisulfite. DNA samples were purified using Wizard DNA purification resin according to the manufacturer's instructions (Promega). These DNA samples were again treated with NaOH, then precipitated with ethanol and resuspended in water. PCR reactions were performed using the primer pairs described below in the following reaction mix: 10× PCR buffer [16.6 mM ammonium sulfate, 67 mM Tris (pH 8.8), 6.7 mM MgCl2, and 10 mM 2-mercaptoethanol], dNTPs (each at 1.25 mM), primers (50 pmol each per reaction), and bisulfite-modified DNA (50 ng) in a final volume of 50 μl. Reactions were hot-started at 95°C for 5 min, after which 1.25 units of *Taq *polymerase (Life Technologies, Inc.) were added. Amplification was carried out in an Applied Biosystem temperature cycler for 40 cycles (30 sec at 95°C, 30 sec at 59°C then 30 sec at 72°C), followed by a final 4-min extension at 72°C. Two sets of primers were used simultaneously to check for methylated or unmethylated CpG islands at the level of hMLH1 and p16 promoters. The primer sequences were as in our previous study [4]. Controls for methylated and unmethylated DNA were DNA from the SW48 colon cancer cell line and normal lymphocytes, respectively. Each PCR reaction product (10 μl) was directly loaded into a 2% agarose gel, which was later stained with ethidium bromide to allow DNA visualization under UV illumination. The presence of a band in unmethylated tumor and matched normal with the absence of a methylation band in the tumor was defined as unmethylated. However, when a methylated band was present for tumor and absent for the normal, we defined the sample as methylated (or semi-methylated if both unmethylated or methylated bands were present in the tumor).

### Histopathological analysis

Independent pathologists who were unaware of the MSI status of any of the samples evaluated specific histopathological characteristics. Grading of tumors was achieved by staining with Hematoxylin-Eosin (H&E). Tumors were classified as proximal (proximal to the splenic flexure) or distal. The TNM system of the International Union against cancer was used for tumor staging. Mucin production was evaluated using the modified criteria of Wiggers et al, [22] and reported as absent (no extracellular mucin production), focal (when extracellular mucin production was present in < 50% of the cells) or predominant (when the area of extra cellular mucin production was present in ≥ 50% of the cells).

### Immunohistochemistry

Tissue obtained from paraffin-embedded blocks was used for the immunohistochemistry experiments. Sections (5 μm) were mounted on charged glass slides, deparaffinized with xylene for 2 × 10 min and rehydrated using a graded ethanol series. Antigen retrieval was performed by placing the samples in a microwave oven for 12 min, with occasional interruption to avoid tissue degradation by excessive heat. The slides were then treated with hydrogen peroxide, followed by incubation with the primary and secondary antibodies, a streptavidin-biotin complex, an amplification reagent, streptavidin-peroxidase and substrate-chromogen solution using the Envision system according to the manufacturers' protocol (DAKO). The samples were then counterstained with hematoxylin, rinsed with ethanol, dried and visualized by a light microscopy. Tissue samples to which no primary antibody had been added were used as negative controls. All immunohistochemistry reagents were purchased from DAKO (Carpinteria, CA) and the antibodies (hMLH1 clone G168-15, 1/100 dilution and hMSH2 clone 556349, 1/500 dilution) were purchased from PharMigen (San Diego, CA). The slides were read by pathologists unaware of the MSI status. Absence of MLH1 and MSH2 nuclear expression was defined as negative staining.

### BRAF mutation analysis

Samples were analyzed for the presence of a point mutation that frequently takes place in the oncogene BRAF, leading to the change of valine into glutamic acid at position 600 of the BRAF protein. DNA from the analyzed samples was used as a template in PCR reactions using two BRAF primers encompassing BRAF exon 15 where the above substitution is known to take place. Amplified fragments were analyzed by sequencing in a 3130 ABI GeneScan to confirm the presence of the mutation.

### Statistical analysis

The MSI phenotypes were divided into two groups: non-MSI (MSS+MSI-L) and MSI-H (two or more markers unstable). Age of patients was a continuous variable, while the stage of the cancer, gender, location, mucin production, differentiation, and hMLH1 and hMSH2 methylation were categorical variables. For statistical analysis, mucin production was categorized as 0 (no production), < 50% or ≥ 50%. The distributions of categorical variables were shown by frequency table, and for age by computing mean (SD). We tested the distribution of categorical variables between MSI-H and non MSI-H groups by asymptotic Chi-Square or the Fisher's-Exact test, as appropriate and computing of OR (95% CI). The age difference between two groups was tested by student *t *test. The univariate analysis was done for both races separately. We selected race, age (as a continuous variable), differentiation, tumor site (right vs. left), stage [1-4], BRAF mutation, hMHL1 and hMSH2 expression (positive vs. negative), and P16 and hMHL1 methylation to enter to a Backward logistic regression model, with MSI as a dependent variable. Univariate analysis showed evidences of interaction between race and some markers, so we developed a model, with interaction terms. Then a final model was developed with the variables with significant effect (p < 0.05) on the risk of MSI-H including interaction term. All analysis was performed by the *SPSS program 15.0 *(Chicago, IL).

## Results

### Distinct clinicopathological features according to MSI status and tumor Site

The clinical and pathological characteristics of the patients are presented in Table 1, 2, 3. For MSI experiments, we analyzed samples from 95 African American patients, (56 females, 39 males). The mean (SD) age of the African American patients (at the time of tissue collection) was 65.7 [15] years. Of the 95 analyzed African American samples, 29 (30.5%) were MSI-H, and 66 (69.5%) were non-MSI-H (Table 1). Four MSI-H patients had a strong family history of colon cancer, and met the Amsterdam criteria for HNPCC. Thirteen other patients met the Bethesda criteria [23], based on the age at diagnosis of CRC; however, tissue samples from thirteen of these patients were non-MSI-H. Sixty-one tissue samples from the Omani patients were analyzed (24 females, 37 males; mean age (SD) was 52.7 (13.6). MSI analysis showed that 53 (86.9%) of Omani samples were non-MSI-H, and 8 (13.1%) were MSI-H (Table 2). From this group, five patients that were MSI-H had a strong family history of colon cancer meeting the Amsterdam criteria for HNPCC. Nineteen other patients met the Bethesda criteria [23], based on the age at diagnosis of CRC; however, tissue samples from fourteen of these patients were non-MSI-H. Fifty-three tissue samples from Iranian patients were analyzed (64% female; mean age (SD) in Iranian patients was 59.8 (12.7; Table 3). Fourteen (26%) of the Iranian samples were MSI-H tumors. The HNPCC and Familial Adenoma Polyposis were not seen in Iranian patients in this study. In the Omani and Iranian populations, there were more MSI-H tumors in males than females (75% vs 25% in Omani; 57% vs. 43% in Iranian); in the African American group, there were more MSI-H tumors in females (66% vs 34%). However, these gender differences were insignificant trends.

**Table 1:** Clinicopathological and genetic characteristics of AA CRC cases

	**African Americans**				
	***All***	***non-MSI-H***	***MSI-H***	**Odds Ratio (95% CI)**	**P**

**Number of patients**	95	66 (69)a	29 (31)a		
**Mean Age**	65.7+15.0 -	65.2+14.0 -	65.9+17.5 -		0.84
**Gender**					0.39
**Female**	56 (59)	37 (57)	19 (66)	1	
**Male**	39 (41)	29 (43)	10 (34)	0.67(0.27–1.67)	
**Site**					0.47
**Distal**	38 (40)	28 (43)	10 (34)	1	
**Proximal**	57 (60)	38 (57)	19 (66)	0.71(0.29–1.77)	
**Mucin Production**	95				0.15
**None**	68 (72)	51(77)	17(58)	1	
**< 50%**	15 (15)	9 (14)	6 (21)	2.0(0.62–6.44)	
**> 50%**	12 (13)	6 (9)	6(21)	3.0(0.85–10.55)	
**Differentiation**	90				0.47
**Well**	3 (3)	2 (3)	1 (3)	1	
**Moderate**	71 (79)	51 (83)	20 (72)	0.64(0.5–8.62)	
**Poor**	16 (18)	9 (14)	7 (25)	0.5(0.17–1.54)	
**Stage**	92				0.59
**Stage 1**	8 (9)	7 (11)	1 (3)	1	
**Stage 2**	37 (40)	24 (38)	13 (45)	0.67(0.09–4.93)	
**Stage 3**	35 (38)	23 (37)	12 (41)	1.08(0.27–4.29)	
**Stage 4**	12 (13)	9 (14)	3 (11)	0.8(0.2–3.27)	
**BRAF**	82				0.00
**wild type**	74 (90)	59 (98)	15 (68)		
**V600E**	8 (10)	1 (2)	7 (32)	27.5(3.1–241.3)	
**hMLH1 methylation (MSP)**	70				0.33
**Unmethylation**	4 (6)	2 (4)	2 (10)	1	
**Methylation**	66 (94)	48 (96)	18 (90)	0.38(0.5–2.87)	
**p16 methylation (MSP)**	47				0.37
**Unmethylation**	28 (60)	14 (54)	14 (67)	1	
**Methylation**	19 (40)	12 (46)	7 (33)	0.58(0.18–1.92)	
**hMLH1 expression by IHC**	76				0.00
**Normal**	50 (65)	45 (83)	5 (23)	1	
**Negative**	26 (35)	9 (17)	17 (77)	16.67(5–50)	
**hMSH2 expression by IHC**	74				0.29
**Normal**	66 (89)	46 (87)	20 (95)	1	
**Negative**	8 (11)	7 (13)	1 (5)	0.33(0.04–2.86)	

**Table 2:** Clinicopathological and genetic characteristics of Iranian CRC cases.

	**Iranian**				
	***All***	***non-MSI-H***	***MSI-H***	**Odds Ratio**	**P**

**Number of patients**	53	39 (74)a	14 (26)a		
**Mean Age**	59.8+12.7 -	59.9+12.7 -	59.4+13.1 -		0.90
**Gender**	53				0.52
**Female**	19(36)	13(33)	6(43)	1	
**Male**	34(64)	26(67)	8(57)	0.67(0.19–2.33)	
**Site**	53				0.00
**Distal**	40(76)	37(95)	3(21)	1	
**Proximal**	13(24)	2(5)	11(79)	66.67(10–500.00)	
**Mucin Production**	NA				
**None**	NA				
**< 50%**	NA				
**> 50%**	NA				
**Differentiation**	53				0.00
**Well**	29(54)	26(67)	3(22)	1	
**Moderate**	22(42)	13(33)	9(64)	6(1.38–26.00)	
**Poor**	2(4)	0	2(14)	NA	
**Stage**	53				0.67
**Stage 1**	0	0	0	NA	
**Stage 2**	12(23)	10(26)	2(14)	1	
**Stage 3**	37(70)	26(67)	11(79)	0.60(0.04–9.16)	
**Stage 4**	4(7)	3(7)	1(7)	1.27(0.12–13.58)	
**BRAF**	53				0.09
**Wild type**	52(98)	39(100)	13(93)		
**V600E**	1(2)	0	1(7)	NA	
**hMLH1 methylation (MSP)**	53				0.00
**Unmethylation**	42(79)	38(97)	4(29)	1	
**Methylation**	11(21)	1(3)	10(71)	95.00(9.53–946.94)	
**p16 methylation (MSP)**	53				0.00
**Unmethylation**	45(85)	38(97)	7(50)	1	
**Methylation**	8(15)	1(3)	7(50)	38.00(4.03–358.74)	
**hMLH1 expression by IHC**	25				0.00
**Normal**	15(60)	12(100)	3(23)		
**Negative**	10(40)	0	10(77)	NA	
**hMSH2 expression by IHC**	25				1.00
**Normal**	24(96)	12(100)	12(92)		
**Negative**	1(4)	0	1(8)	NA	

**Table 3:** Clinicopathological and genetic characteristics of Omani CRC cases.

	**Omani**				
	***All***	***non-MSI-H***	***MSI-H***	**Odds Ratio**	**P**

**Number of patients**	61	53 (87)a	8 (13)a		
**Mean Age**	52.7+13.6 -	53.1+12.8 -	51.5+19.2 -		0.76
**Gender**					0.37
**Female**	24 (39)	22 (42)	2 (25)	1	
**Male**	37 (61)	31(58)	6 (75)	2.12(0.39–11.55)	
**Site**	61				0.75
**Distal**	51 (84)	44(83)	7(87)	1	
**Proximal**	10 (16)	9(17)	1(13)	1.43(0.16–13.11)	
**Mucin Production**	61				0.44
**None**	47 (77)	42(78)	5 (63)	1	
**< 50%**	14(23)	11 (20)	3(37)	2.3(0.47–11.10)	
**> 50%**	0	0	0	NA	
**Differentiation**	60				0.44
**Well**	7 (12)	7 (14)	0	NA	
**Moderate**	49(82)	43 (81)	6(86)	1	
**Poor**	4 (6)	3 (6)	1 (14)	2.39(0.21–26.84)	
**Stage**	61				0.28
**Stage 1**	1 (2)	1 (2)	0	NA	
**Stage 2**	20 (33)	15 (28)	5 (62)	1	
**Stage 3**	31 (50)	29 (55)	2 (25)	0.21(0.02–1.50)	
**Stage 4**	9 (15)	8 (15)	1 (13)	0.38(0.01–4.41)	
**BRAF**	59				0.62
**Wild type**	48 (81)	42 (82)	6 (75)	1	
**V600E**	11 (19)	9 (18)	2 (25)	1.56(0.27–9.0)	
**hMLH1 methylation (MSP)**	49				0.47
**Unmethylation**	8 (16)	6(14.6)	2 (25)	1	
**Methylation**	41 (84)	35 (85.4)	6 (75)	0.51(0.08–3.17)	
**p16 methylation (MSP)**	44				0.09
**Unmethylation**	27(62)	20 (56)	7 (88)	1	
**Methylation**	17 (38)	16 (44)	1 (12)	0.18(0.02–1.61)	
**hMLH1 expression by IHC**	61				0.68
**Normal**	42 (69)	37(70)	5 (62)	1	
**Negative**	19 (32)	16 (30)	3(38)	1.39(0.29–6.67)	
**hMSH2 expression by IHC**	61				0.63
**Normal**	56 (92)	49 (92)	7(87)	1	
**Negative**	5 (8)	4 (8)	1(13)	1.75(0.17–16.67)	

Parentheses indicate percentages: All values are based on column except _a _based on row. MSP = methylation-specific PCR; IHC = immunohistochemistry

A total of 57 (60%) primary African American tumors were located in the proximal colon and 40% [38] were distal. Within the MSI-H group, 19 (66%) were proximal and 10 (34%) were distal (Table 1). The non-MSI-H group showed a distribution with 38 (57%) proximal tumors and 28 (43%) distal tumors. Mucin production was noted in 27 tumors including 12 MSI-H and 15 non-MSI-H tumors. Fifty-one of the Omani tumors were located in the distal colon (84%). The prevalence of distal lesions in the MSI-H group was 87%. Of the 61 Omani samples, 47 (77%) showed no mucin production, 14 (23%) had focal mucin production (Table 2). The percentage of lesions that produced mucin was 20% and 37% for MSI-H and Non-MSI-H, respectively. Frequency of proximal tumors in Iranian patients was 13 (24%) and the prevalence of proximal lesions in the MSI-H group was 79% [11]. In univariate analysis, a proximal location increases the rate of MSI-H in Iranian patients significantly (OR = 66.67(10–500)) (p = 0.001; Table 3)).

The majority of tumors were found to be moderately differentiated in African Americans (79%) and Omanis (82%; Table 1, 2). Iranian tumors were mostly well differentiated (54%). The percentage of moderately differentiated tumors was 86% in the MSI-H Omani group compared with 72% in African Americans and 64% in Iranians (Table 1, 3). In addition, seven and one MSI-H tumors showed poor differentiation in African Americans and Omanis, respectively. While in African Americans, one MSI-H was well differentiated, no patient in Iranian non-MSI-H cases was poorly differentiated (Table 1, 3). The difference was insignificant trend in African Americans and Omanis but in Iranian patients differentiation was significantly correlated with MSI-H occurrence. This finding was not sustained in multivariate analysis.

Most of the patients in the series were stage II or higher (Table 1, 2, 3), with 45% [13] of African American, 62% [5] of Omani, and 14% [2] of Iranian MSI-H lesions at stage II. Thirty-eight percent [24] of African American, 28% [15] of Omani, and 23% [12] of Iranian of the non-MSI-H lesions were at stage II (Table 3). Forty-one percent [12] of African American, 25% [2] of Omani, and 79% [11] of Iranian of the MSI-H lesions were at stage III (Table 1, 2, 3). Thirty-seven percent [23] of African American, 55% [29] of Omani, and 67% [26] of Iranian of the non-MSI-H lesions were at stage III. Fourteen percent [9] of African American, 15% [8] of Omani, and 7% [3] of Iranian of the non-MSI-H lesions were at stage IV (Table 1, 2, 3). There was insignificant trend in the stage of the lesions between the MSI and non-MSI-H groups.

### Correlation between methylation pattern of the hMLH1 promoter and MSI status

Among MSI-H samples, hMLH1 promoter methylation was found in 90% [18] of African American, 75% [6] of Omani and 71% [10] of Iranian tumors, confirming a previous finding of predominant methylation of these genes of the promoter in Africans (Table 1, 2, 3) [3,4]. Non-MSI-H tumors in both African Americans and Omani tumors showed methylation of the hMLH1 promoter as well (> 80%). In Iranian non-MSI-H patients, the frequency of hMLH1 methylation was 3% [1]. The methylation status of hMLH1 does not seem to be gender- or stage-specific. In multivariate analysis, including the interaction terms, the hMLH1 methylation has a significant effect on MSI-H occurrence (OR = 13.84(1.05–182.72); p = 0.046) (Table 4). In MSI-H tumors, p16 methylation occurred in 33% [7] of African American, 12% [1] of Omani and 50% [7] of Iranian tumors. While it was more than 40% in non-MSI-H tumors in African Americans and Omani patient populations, this figure was 3% [1] in Iranian non-MSI-H CRC patients.

**Table 4:** Multivariate analysis for the risk factors associated with MSI-H occurrence (Model including single term and interactions effect)

Factor	OR (95% CI)	P value
Normal hMLH1 expression by IHC	0.13(0.04–0.41)	< 0.0001
BRAF V600E mutation	12.11(2.82–52.03)	0.001
hMLH1 methylation	13.84(1.05–182.72)	0.046
Race		0.946
African-Americans	1	
Omani	0.83(0.04–0.18.30)	0.905
Iranian	0.68(0.07–6.58)	0.739
hMLH1 methylation* Race		0.072
hMLH1 methylation* Omani	0.04(0.01–1.74)	0.091
hMLH1 methylation* Iranian	0.02(0.01–0.63)	0.032

* indicates the interaction term between two variables.

### Correlation between hMLH1 protein expression and MSI status

Expression of the hMLH1 and hMSH2 nuclear proteins was examined immunohistochemically in paraffin-embedded tissue sections [4]. In all cases, non-neoplastic cells displayed positive nuclear staining for both hMLH1 and hMSH2. The nuclear stained slides were read by two different pathologists, and normal tissue on slides where no primary antibody stained was used as negative control. In total, 76 African American, 61 Omani, and 25 Iranian cases were analyzed. Thirty-five and 11% of African American tumors assayed showed loss of staining for hMLH1 and hMSH2, respectively. Moreover, loss of hMLH1 and hMSH2 staining was observed in 77% and 5% of MSI-H African American tumors, respectively (Table 1). The loss of hMLH1 and hMSH2 staining was observed in 17% and 13% of non-MSI-H African American tumors, respectively (Table 1). In Omani tumors: 38% (n = 3) of the MSI-H tumors showed absences of hMLH1 expression (Table 2); 30% (n = 16) of the non-MSI-H tumors had negative hMLH1. Fifty-six cases of Omani patient tested for the presence of hMSH2 protein by immunohistochemical staining, seven Omani cases were MSI-H. In Iranian patients, 77% [10] of the MSI-H tumors were negative for hMLH1 expression (Table 3); all of non-MSI-H tumors were positive for hMLH1 expression, while for hMSH2 these figures were 8% [1] and 0, respectively. In multivariate analysis, including the interaction terms, the hMLH1 expression has a significant preventive effect on MSI-H occurrence (OR = 0.13(0.04–0.41); p < 0.0001; (Table 4)).

### Correlation between BRAF mutation and MSI status

Samples were analyzed for the presence of a point mutation that frequently occurs in the oncogene BRAF leading to the change of valine to glutamic acid at position 600 of the BRAF protein. In all samples only 10% [8] African American, 19% [11] Omani and 2% [1] Iranian samples displayed the mutation by sequencing (Table 1, 2, 3). In the MSI-H group, the BRAF mutation was 32% [7] in African Americans, 25% [2] in Omani, and 7% [1] in Iranian, respectively. Eighteen percent and 2% of non-MSI-H tumors carry the BRAF mutation in African Americans and Omanis, respectively, while none of the Iranians carried a BRAF mutation in this group. A BRAF mutation is a significant risk factor for MSI-H occurrence even after controlling for other factors in the multivariate model (OR = 12.11(2.82–52.03); p = 0.001; (Table 4)).

## Discussion

DNA MMR gene silencing and/or mutation are the main factors leading to accumulation of mutations within genes and general genome instability [19]. The MSI phenotype resulting from such alterations is one of the early events leading to the development of certain cancers. It occurs in patients with Lynch syndrome and in those that acquire such alterations somatically [24]. Another event occurring in adenoma polyp is mutation in oncogenes, specifically in K-ras and BRAF genes in the case of colon cancer [24-26]. The linkage between BRAF mutations and MSI was previously reported [27-30]. In general, Omani people have a mixed Asian and African ethnic background [10] and Iranians are of Caucasian ethnicity. Here we demonstrate that in African Americans, Omanis and Iranians, the MSI phenotype occurs in 31%, 26% and 13% of CRC tumors, respectively. In addition, the locations of the tumors in African Americans and Iranians were primarily proximal compared to distal for Omanis; and BRAF mutations were more prevalent in Omanis than African Americans or Iranians (19%, 10% and 2%, respectively). While the MSI phenotype in this sample of African Americans is less than we previously reported (43–45%), [3,4] it appears to be more prevalent in African Americans than in others and still high in the general US population [31-33]. The current sample contained more African Americans and more right-sided than left-sided tumors. The MSI phenotype was more common in proximal tumors than in distal ones (p = 0.47), strengthening earlier findings of such an association [31-33]. The prevalence of MSI-H tumors indicates the importance of BRAF mutations, particularly in the African Americans, since they represent a higher mutation profile than Iranians and Omanis. This finding suggests that, at least within the MSI-H group of patients, the BRAF V600E mutation in exon 15 is the major event leading to tumor development. In addition, the distal location of Omani MSI-H tumors may also play an important role in the presentation of the disease compared to proximal ones in the African American and Iranian populations. The lower frequency of MSI-H tumors observed in Omani patients could be related to the higher frequency of the distal location. This needs to be verified in a larger sample size.

The absence of hMLH1 expression was more pronounced in MSI-H tumors from African American and Iranian patients (77% of tumors in these groups showed no hMLH1 expression). However, for Omanis, the moderate hMLH1expression (62%) in MSI-H may indicate some other defects in the component of base excision repair (Oxidative DNA damaged markers such as 8-hydroxyguanosine), or distinct lymphangiogenic phenotype associated with MSI other than in mismatch repair genes. The tumors that we have studied included MSI-H tumors that were negative using immunohistochemistry for hMLH1. This incidence is less than compared to that described in the literature (77% [34]), although the numbers described are small.

These results are in concordance with the MSP analysis showing that a majority of samples displayed methylation at the hMLH1 promoter. The methylation of hMLH1 is most likely from a specific genome methylation process in progression of colon cancer. The African American group was older than both the Omani and Iranian group. It has been demonstrated that older people, particularly those in the 7th–9th decade of life, have a much higher chance of hypermethylation of hMLH1 and MSI. This is consistent with hMLH1 methylation in the African American cancers both in MSI-H and non-MSI cases. However, in Iranians, a higher profile of epigenetic silencing of hMLH1 may explain the MSI-H tumors but not in non-MSI. In addition, the distal location of Omani tumors may also play an important role in the presentation of the disease compared to the African American who has generally proximal CRC.

Our results demonstrate that methylation of hMLH1 is the major cause of MSI in sporadic CRC consistent with our previous findings [3,4]. The fact that the hMLH1 gene is methylated indicates that it may be inactivated by an epigenetic mechanism. The association of higher levels of CpG island methylation with more advanced histological changes suggests that CpG methylation plays a role in CRC [30]. However, the pathophysiology of hyper-methylation (the why, when and where) has yet to be elucidated. Cancers can be classified according to their degree of methylation. Those with high degrees of methylation (the CpG island methylator phenotype, or CIMP) represent a clinically and etiologically distinct group that is characterized by 'epigenetic instability'. The MSI-H and CIMP phenotype may explain the criteria in proximal tumors in African American and Iranian but not the distal ones in Omani tumors; however, the CpG island methylation status of a broader panel of genes needs to be investigated to determine whether this is the case. Some samples for MLH1 methylation failed to display MLH1 protein by immunohistochemistry and were non-MSI (MSS and MSI-L). Possible explanations might be that the targeted CpG island for methylation in this study is located upstream of the gene, and has a minor effect on the transcription of MLH1 protein [35] or that the detected methylation is only in a small cell population that does not reflect the overall tumor phenotype. Full methylation of the hMLH1 promoter region and subsequent gene inactivation may play a crucial role in carcinogenesis of MSI-H CRCs. Therefore, in our on-going investigation, we are studying the methylation status of the lesions by examining all CpG sites [36] especially within non-MSI tumors where data reveals lack of protein expression. Some non-MSI tumors were methylated at the MLH1 site and this may be due to the partial or hemi-methylation at an altered MLH1 site. This is consistent with the lack of correlation of between the methylation status and level of expression of MLH1[37]. Indeed, more markers need to be considered to measure methylation in order to establish a methylation phenotype that correlates the protein expression status and allows for the understanding of preferential carcinogenic pathways. Positive staining was confirmed in adjacent normal tissue within the same slide validating of the staining for specimens that were found to be negative for either MLH1 or MSH2. In addition, preferential microsatellite loci containing large repeat units, but not loci containing mono- or dinucleotide repeats units, may contribute to the non-MSI tumors, particularly MSI-L CRC tumors.

Many explanations could account for the differences between African Americans and the other populations. The difference in MSI frequency might be due to genetic specificities or to behavioral/dietary causes [38]. Dietary factors such as folate, vitamins, and methionine may be associated with colon cancer because of involvement in DNA methylation and hence on the CpG island, MSI and BRAF. A recent study questioned the unique role for dietary folate, alcohol, vitamins B_6 _and B_12 _and methionine in the CIMP phenotype [39]. In addition, use of alcohol and obesity were associated with an increased risk of tumors that were MSI-H and CIMP-low (less than two markers methylated) [39]. The number of lymph nodes, distance organ metastases and additional impairment of the MMR system may associate with the more aggressive behavior of CRC in African Americans.

Studies have investigated the traditional (nonserrated) adenoma-carcinoma sequence and the serrated polyp neoplasia for BRAF and MSI. MSI-H was identified only in the adenocarcinoma component of serrated carcinomas [40,41]. BRAF mutation has been shown to be a specific marker for a serrated polyp pathway that has its origin in a hyperplastic polyp and a potential end point as MSI carcinoma. A recent study indicated that MSI-H in the sporadic colorectal cancers may be part of a clinically distinct subgroup with a high incidence of BRAF mutation developed from serrated polyps [26]. In this study we categorized the histological status of the end point such as carcinomas with residual adenoma, the serrated polyp neoplasia pathway and the traditional (nonserrated) adenoma-carcinoma sequence. However, we did not find any evidence of serrated or serrated hyperplastic tissue in the MSI-H tumors. Therefore, our BRAF and MSI-H analyses indicate that tumors may not belong to the serrated pathway, which needs additional investigation. Most hyperplastic polyps occur in the rectum and sigmoid colon, but most serrated (hyperplastic) polyps occur in the right colon and are associated with proximal cancers [40,41]. All but one (13%) Omani, 79% Iranian, 66% of African American MSI-H tumors were proximal with no evidence of serrated hyperplastic polyps. Tumors in these populations were moderately differentiated with mixed adenomas. Therefore, at this time it is not clear whether or not mixed adenoma types have any role in the MSI-H in African Americans.

The population of the Sultanate of Oman is especially interesting because it represents a combination of African and Asian heritage in a small country with the distribution of CRC disease in young people, which may be due to the age structure of the population in Oman. The effect of environmental factors such as diet, physical activity, access to health care, frequent tribal marriage and lifestyle can not be ruled out for the occurrence of the CRC in young age [9,10,42,43]. The Iranian population is a large collection of ethnic groups and their descent were from ancient Iranian peoples[44]. Modern Persians themselves are also a heterogeneous group of peoples descended from various Caucasian peoples [14,45]. The younger age distribution of Iranian CRC disease also may be due to the age structure of the population. However, the effect of environmental factors such as diet and lifestyle can not be ruled out. Our data on CRC lead us to speculate the complex interactions of genotype, environmental factors (such as diet), and other lifestyle factors in the pattern of bowel cancer. Further epidemiology data to compare the age-specific incident rates for Oman, Iran and African American is needed to confirm this finding.

The proximal location of tumors in African Americans is consistent with the tumors in Western and Asian studies [31-33,46,47] which may reflect the impact of western diets on the African Americans. However, the high MSI level for African Americans for this limited sample size may in part be due to the increased age of these patients along with finding the majority of the tumors on the right side of the colon. The Iranian tumors closely mimic what we see in Caucasians in the U.S., most tumors are left sided, most of the MSI-H tumors are right sided and the MSI-H tumors are associated with higher tumor differentiation. The distal location of the Omani tumors and the low MSI level is an important observation compared to the moderate and high proximal MSI level in Iranian and African American tumors, respectively. The alteration of K-RAS may also contribute to the methylator phenotype in CRC, especially in the Iranian tumors, which we plan to study in the future [30]. The rate of mutation and its level in MMR deficiency may contribute and determine the frameshift rate for the loss of these proteins in CRC, hence different behavior from these tumors.

All of these factors may contribute to the risk of colon cancer and presentation of the disease in African American, Omani and Iranian patients. We cannot rule out a genetic predisposition in these patients since we did not have access to detailed family history and, therefore, some of the subjects may have had genetic and epigenetic predispositions. In this study we did not use other groups such as white Americans, other Africans and Asian in our controls for any direct or indirect comparison analysis. We plan to have a more detailed study regarding the diversity of these populations since the scope of this study focused on the clinicopathological features.

In conclusion, this first comparative clinicopathological investigation in three different populations suggests that the MSI-H CRC phenotype in African American, Iranian and Omani patients is significantly associated with BRAF mutation and hMLH1 expression. CRCs in African Americans tend to be higher in microsatellite instability (32%) and more often located in the proximal colon, compared to Iranian and Omani CRC tumors. The high level of MSI-H in African Americans may have significant implications in treatment plans, because MSI-H lesions are often right-sided and may show a different response to chemotherapeutic agents such as 5-fluorourcil [48].

## Competing interests

The authors declare that they have no competing interests.

## Authors' contributions

HB carried out the MSI study. PM, FN, and MS recruited Iranian samples and run MSI and immunohistochemistry. MA, KAl-M, RAl-M, and AAl-S, and SR recruited Omani samples from different hospital in Oman and run methylation analysis. FG recruited samples from Johns Hopkins and performed the H&E staining. DTS help in the sample recruitment and reviewing the data. from Howard university. AV, RCB and AG performed run the BRAF mutation analysis. EL, MH performed the pathological analysis, MN run the statistics. HA designed the experiment and data analysis. All authors read and approved the final manuscript.
